# The use of new technology in prevention and treatment of psychiatric diseases - preliminary results

**DOI:** 10.1192/j.eurpsy.2023.1808

**Published:** 2023-07-19

**Authors:** A. Häussl, F. Fellendorf, E. Fleischmann, S. Guggemos, E. Schönthaler, T. Stross, I. Zwigl, D. Albert, J. Mosbacher, K. Stix, S. Draxler, G. Lodron, T. Orgel, M. Pszeida, S. Russegger, M. Schneeberger, M. Uray, W. Weiss, M. Fellner, T. Fruhmann, R. Hartmann, P. Hauptmann, R. Pfiszter, G. Pötz, U. Prattner, N. Saran, S. Spat, E. Zweytik, T. Lutz, S. Lindner-Rabl, R. Roller-Wirnsberger, S. Schüssler, J. Zuschnegg, K. Ceron, M. Danilov, C. Grossegger, M. Macher, O. Sokolov, S. Egger-Lampl, B. Roszipal, L. Paletta, M. Lenger, N. Dalkner, E. Reininghaus

**Affiliations:** 1 Clinical department of Psychiatry and Psychotherapeutic Medicine, Medical University of Graz; 2 Institute of Psychology, University of Graz; 3 DIGITAL – Institute for Information and Communication Technologies, Joanneum Research Forschungsgesellschaft mbH; 4 digitAAL Life GmbH, Graz; 5 Symptoma GmbH, Attersee; 6 Department of Internal Medicine, Research Unit Aging and Old Age Medicine, Medical University of Graz, Graz; 7 Humanizing Technologies, Vienna; 8 MINDCONSOLE GmbH, Graz, Austria

## Abstract

**Introduction:**

The COVID-19 outbreak is a serious global public health issue with wide-ranging negative effects on people’s lives, which is reflected in steadily rising mental health problems. In order to appropriately respond to the increased occurrence of psychiatric illness, protect mental health and strengthen resilience it is necessary to include new technologies, such as extended reality (XR) or socially assistive robots (SAR) in not only psychiatric treatment but also in the prevention of psychiatric diseases. In this context, the use of new technologies offers innovative ways to strengthen resilience, self-efficacy and stress coping skills and plays an important role in improving psychological wellbeing.

**Objectives:**

Preliminary results from studies at the Clinical Department of Psychiatry and Psychotherapeutic Medicine in Graz, Austria, dealing with new technologies in psychiatry, show new options for psychiatric settings.

**Methods:**

Project **AMIGA:** The aim of this study is to test the effectiveness of a cognitive training session, conducted with the SAR named Pepper. In this randomized controlled trial, the effectiveness of SAR on depressive symptoms and correlates is evaluated in a sample of 60 individuals with major depression. While the intervention group will receive cognitive training with the SAR Pepper, the control group will receive “treatment-as-usual” therapy with a common PC software. Participants will receive 30 minutes of training 2 times per week over a period of 3 weeks.

Project **XRes4HEALTH:** The aim of this study is to develop an XR resilience training to increase resilience and stress coping mechanisms in healthcare workers. A total of 40 people will be included. To test the effectiveness of the resilience training, 3 XR training sessions of 15 minutes each will be held. A pre-post measurement will test the effectiveness of the training on wellbeing and stress levels as well as the acceptance and satisfaction with the training.

Project **AI-REFIT:** The overall goal of this study is to explore key information to increase resilience in healthy individuals who are at increased risk for mental health problems. Through a usability study, the artificial intelligence-based prototype app of the resilience training will be tested for acceptance, usability, functionality, and efficiency. During the resilience training, participants are wearing a smartwatch which measures psychophysiological parameters. Conclusions about the success of the therapy can be drawn based on digital data acquisition.

**Results:**

New technologies including XR and SAR support classical psychiatric treatment in the topics of resilience and cognitive training as an add-on therapy in times of reduced availability of healthcare workers.

**Conclusions:**

The rapid development of new technologies holds a lot of potential in the treatment of psychiatric disorders, which is why it is important to scientifically evaluate those innovative tools.

**Disclosure of Interest:**

None Declared

